# Supplementary material for: A Humanized Yeast Phenomic Model of Deoxycytidine Kinase to Predict Genetic Buffering of Nucleoside Analog Cytotoxicity
Source: Genes (Basel). 2019 Sep 30;10(10):770. doi: 10.3390/genes10100770 (PMC6826991; doi:10.3390/genes10100770)
Supplement: Supplementary file 1 [file genes-10-00770-s001.zip › Additional_Files/Additional_File7_GOTermHeatmaps.zip!/Additional_File7_GOTermHeatmaps/polysome.pdf]

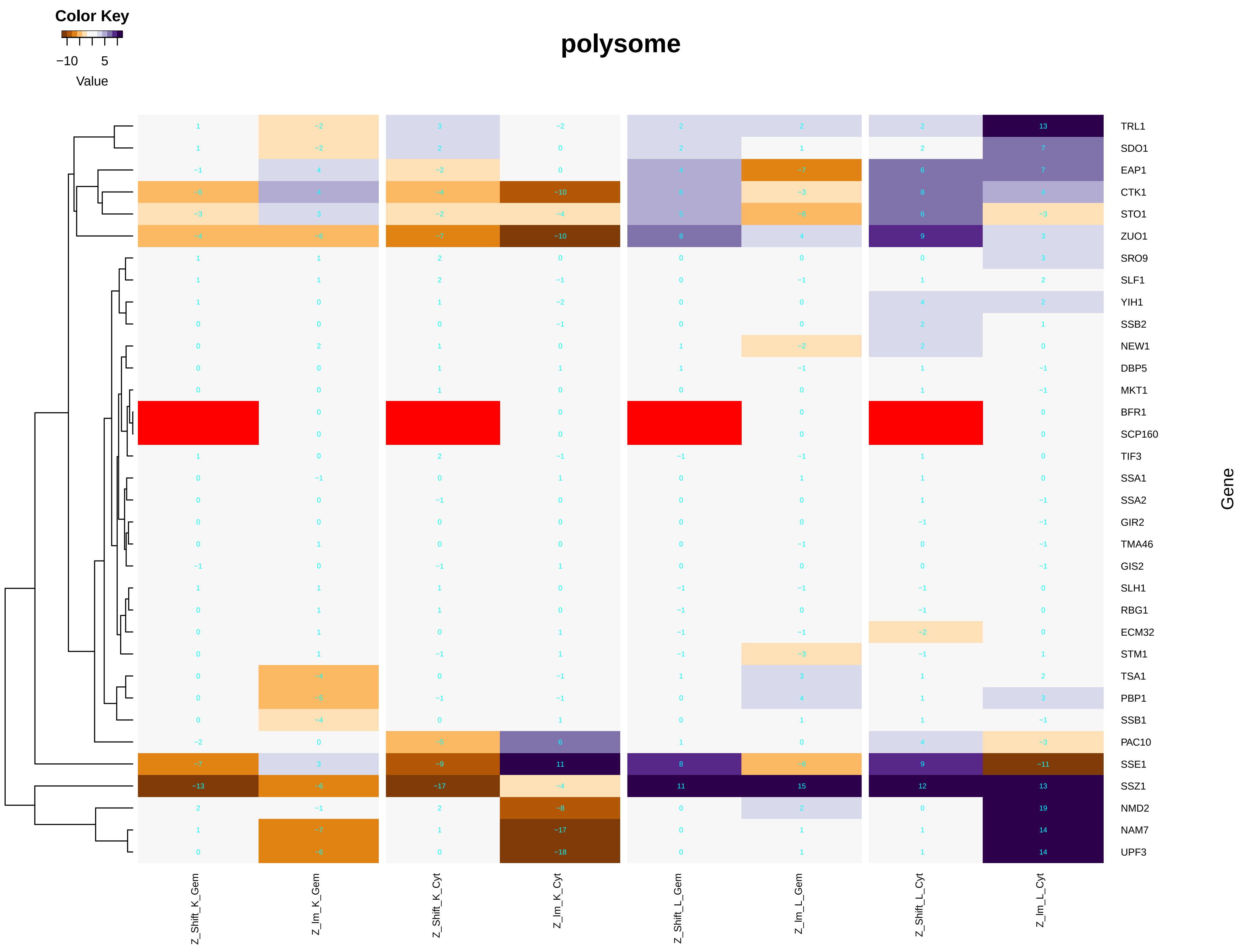

Color Key
−10 5
Value
polysome
TRL1
SDO1
EAP1
CTK1
STO1
ZUO1
SRO9
SLF1
YIH1
SSB2
NEW1
DBP5
MKT1
BFR1
SCP160
TIF3
SSA1
SSA2
GIR2
TMA46
GIS2
SLH1
RBG1
ECM32
STM1
TSA1
PBP1
SSB1
PAC10
SSE1
SSZ1
NMD2
NAM7
UPF3
Gene
Z_Shift_K_Gem
Z_Im_K_Gem
Z_Shift_K_Cyt
Z_Im_K_Cyt
Z_Shift_L_Gem
Z_Im_L_Gem
Z_Shift_L_Cyt
Z_Im_L_Cyt
